# Towards reporting guidelines of research using whole-body vibration as training or treatment regimen in human subjects—A Delphi consensus study

**DOI:** 10.1371/journal.pone.0235905

**Published:** 2020-07-22

**Authors:** Anika Wuestefeld, Anselm B. M. Fuermaier, Mario Bernardo-Filho, Danúbia da Cunha de Sá-Caputo, Jörn Rittweger, Eckhard Schoenau, Christina Stark, Pedro J. Marin, Adérito Seixas, Stefan Judex, Redha Taiar, Csaba Nyakas, Eddy A. van der Zee, Marieke J. G. van Heuvelen, Oliver Tucha

**Affiliations:** 1 Department of Clinical and Developmental Neuropsychology, University of Groningen, Groningen, Netherlands; 2 Laboratory of Mechanical Vibration and Integrative Practices, Universidade do Estado do Rio de Janeiro, Rio de Janeiro, Brazil; 3 Faculty Bezerra de Araújo, Physiotherapy Course, Universidade do Estado do Rio de Janeiro, Rio de Janeirom, Brazil; 4 German Aerospace Center, University of Cologne, Cologne, Germany; 5 Department of Pediatrics, Medical Faculty and University Hospital, University of Cologne, Cologne, Germany; 6 Center for Prevention and Rehabilitation, UniReha GmbH, Medical Faculty and University Hospital, University of Cologne, Cologne, Germany; 7 CyMO Research Institute, Valladolid, Spain; 8 Escola Superior de Saúde, Universidade Fernando Pessoa, Porto, Portugal; 9 Department of Biomedical Engineering, Stony Brook University, Stony Brook, NY, United States of America; 10 University of Reims, Reims, France; 11 Department of Morphology and Physiology, Faculty of Health Sciences, Semmelweis University, Budapest, Hungary; 12 Department of Molecular Neurobiology, University of Groningen, Groningen, Netherlands; 13 Center for Human Movement Sciences, University of Groningen, University Medical Center Groningen, Groningen, Netherlands; University of L'Aquila, ITALY

## Abstract

**Background:**

Whole-body vibration (WBV) is a method utilizing vibrating platforms to expose individuals to mechanical vibration. In its various applications, it has been linked to improved muscular, skeletal, metabolic, or cognitive functioning, quality of life, and physiological parameters such as blood pressure. Most evidence concerning WBV is inconclusive and meta-analytical reviews may not readily produce insights since the research has a risk of misunderstandings of vibration parameters and incomplete reporting occurs. This study aims at laying an empirical foundation for reporting guidelines for human WBV studies to improve the quality of reporting and the currently limited comparability between studies.

**Method:**

The Delphi methodology is employed to exploit the integrated knowledge of WBV experts to distil the specific aspects of WBV methodology that should be included in such guidelines. Over three rounds of completing online questionnaires, the expert panel (round 1/2/3: 51/40/37 experts respectively from 17 countries with an average of 19.4 years of WBV research experience) rated candidate items.

**Results:**

A 40-item list was established based on the ratings of the individual items from the expert panel with a large final consensus (94.6%).

**Conclusion:**

The final consensus indicates comprehensiveness and valuableness of the list. The results are in line with previous guidelines but expand these extensively. The present results may therefore serve as a foundation for updated guidelines for reporting human WBV studies in order to improve the quality of reporting of WBV studies, improve comparability of studies and facilitate the development of WBV study designs.

## Introduction

Whole-Body Vibration (WBV) has received increasing attention in the field of physical, physiological, and psychological rehabilitation [1–3]. WBV is a method utilizing vibrating platforms (i.e., training devices) to expose the individuals’ whole body to vibration (usually with a frequency between 5–60 Hz, depending on the device). This technique was originally aimed at improving physical, muscular, and skeletal functioning [4–6], but has been employed for more widespread applications since. So, the focus lies on WBV as a training or treatment method, which must be distinguished from detrimental vibrations that people may be exposed to during transportation or in occupational settings [7].

In its original application, the short- and long-term effects on physical functioning within different scientific fields including medicine and movement sciences were examined [8–10]. More recently, WBV was also evaluated for its effects on, among others, blood pressure [11], body mass [12,13], pulmonary function [14], quality of life [15] and cognitive functioning [16–18]. WBV research in clinical settings is complemented by evidence from animal studies [19–22]. With these new applications, the field of WBV research became more extensive, leading to (1) an increasing number of studies, (2) new and additional outcome measures, (3) further methodological differences regarding devices, vibration parameters and research designs used [23], and ultimately, (4) growing interdisciplinarity.

Across all applications of WBV in the various research fields, the evidence concerning its effects on various functions has been inconsistent (e.g., see these reviews [6,8,10,24–29]). Based on the inconclusive evidence within the different fields, no firm conclusions about the effects of WBV training machines can be drawn as of now. More high-quality studies with improved methodology [30] and complete and correct reporting with sufficient detail may help to receive greater insight into the effects of WBV. Such studies can form the basis for future meta-analytic studies [31] in order to identify modulating factors of WBV effects and, ultimately, to identify optimal settings.

WBV regimes are defined by several technical components. Essential vibration parameters include, but are not limited to, vibration frequency, amplitude or acceleration, and duration [1,6,23,32]. These parameters are commonly reported inaccurately or completely omitted from study reports [32] and can be subject to misunderstandings because of inconsistent terminology (e.g. amplitude vs. peak-to-peak amplitude vs. peak-to-peak displacement) and lack of information how these parameters were assessed [1,32]. Thus, misunderstandings or omissions may not be readily apparent and may be included in published work. Based on the interdisciplinarity of WBV research, coherent terminology may be difficult to attain [32]. Yet, omitting important information or using vibration-specific terms interchangeably may lead to inaccurate conclusions and will preclude high-quality study designs, their replicability, the reporting of results, the comparability between studies, and the ability to explain inconsistencies [1]. Therefore, complete and correct use of terminology is essential to ensure high-quality reporting, as well as increasing the possibility and validity of comparing findings (meta-analyses or systematic reviews). The possibility that these consistencies lead to the incomparability of studies urges the consideration of consistent reporting.

As a consequence of these issues and to achieve a deeper understanding of the possible effects of WBV, complete, consistent, and correct reporting is vital. A common manner to ensure consistent reporting is the adherence to standardized reporting of research. The EQUATOR (Enhancing the QUAlity and Transparency Of health Research) Network [33] provides reporting guidelines such as the Preferred Reporting Items for Systematic Reviews and Meta-Analyses (PRISMA) or the Consolidated Standards of Reporting Trials (CONSORT) statement [34,35]. These statements identify key characteristics of any high-quality publication.

Adherence to already existing general reporting guidelines, like CONSORT, is not sufficient to ensure high-quality reporting in the field of WBV since the guidelines are not specific enough to ensure complete, consistent, and correct reporting. The inherent technical components of WBV and their susceptibility to misunderstandings, the large variation in devices, parameter settings, and its applications require tailored guidelines. Therefore, instead of following only the CONSORT statement, new guidelines applicable to WBV research should be adopted. Ideally, these guidelines will function as an addition to the CONSORT statement and apply to all research utilizing WBV.

Previous efforts towards developing reporting guidelines specific to WBV research specified reporting recommendations for WBV studies and defined and explained the physical principles of WBV (Rauch and colleagues [1], representing the International Society of Musculoskeletal and Neuronal Interactions). The recommendations were based on suggestions by experts, but suggestion collection and the decision process behind selecting suggestions for the recommendations are unclear. In addition, recent developments in the field of WBV research (e.g., new outcome measures, changes of devices, animal or cell culture studies) are not incorporated.

The EQUATOR Network published recommendations on establishing new reporting guidelines [36] and provides a platform for guidelines to increase their impact. In order to encourage the development of guidelines when needed, the Network recommends that reporting guidelines should (1) include a range of stakeholders with expertise, (2) generate a list of items through, for example, a Delphi consensus study, followed by (3) meetings of the executive group to discuss items or additional information which should be included [36].

Unlike prior publications initiating reporting guidelines or explanations of terminology [1,6,32], systematically obtained consensus between WBV experts about the crucial aspects of reporting human WBV research can lay the foundation for reporting guidelines. These guidelines should be widely accepted by the scientific community, can be used in all disciplines using WBV, and follow the recommendations of the EQUATOR Network. Thus, the aim of the present study is to commence the development of such reporting guidelines. The aim is *not* to collect aspects of studies relevant to reporting research which are outlined in general reporting guidelines (e.g., the CONSORT statement) not specific for WBV research (e.g., sample size, age of participants, type of study). The final reporting guidelines, which will be predominantly based on the present study and finalized by the executive group, will act as an addition to these general reporting recommendations. An overview of the background of the 14 members of the executive group can be found in the supporting information (S1 File).

The Delphi consensus methodology is utilized to inquire and collect the opinion and knowledge of experts in the field from various backgrounds in the present study. This complies with the recommendations of the EQUATOR Network, is a first step in the process of developing reporting guidelines for WBV research on humans, and uses the prior publication by Rauch et al. [1] as a starting point.

## Method

### Delphi consensus methodology

The present study utilized the Delphi method. This method is a structured process to gather information about a certain topic from a relevant group of people or experts [37]. It is based on the assumption that the combination of opinions from various individuals lies closer to the truth than the opinion of one person alone [38]. The Delphi method generally includes several rounds during which the opinion of experts is inquired in a structured, yet interactive, manner [39]. Throughout these rounds, controlled feedback is given to gain the most reliable consensus of a panel of experts. This allows re-evaluation of prior responses and is thought to produce trustworthy and reliable information. Building on the knowledge of the participants, the result reflects their insights and opinions [40].

For the purposes of this study, a modified Delphi method of three rounds was employed to gain an understanding about which aspects should be included in reporting guidelines for human WBV studies. An overview of the rounds is given in Table 1 and the full instructions and questionnaires can be found in the supporting information (S2 File). If necessary, more rounds would be added during the progress of the study. Instead of the traditional Delphi approach of starting the first round with open-ended questions [41], the first round in the present study was modified to begin with a pre-selected list of items. It has been deemed acceptable to modify the first round if it is based on literature and prior knowledge; is a common approach to the first round [41,42] and may increase response rate [43]. The This list of items in the present study was constructed by means of a literature review, the previous recommendations by Rauch et al [1] and related publications [6,23,32], experience of the authors, and a discussion during the second conference of the World Association of Vibration Exercise Experts (WAVEX; August 2018, Groningen, The Netherlands).

**Table 1:** Overview of the Delphi rounds for the establishment of reporting guidelines in human WBV studies.

Round 1	• Panel members indicate whether pre-selected items of importance should commonly be reported
	• Panel members suggest additional items of importance
Round 2	• Panel members receive structured feedback of the previous round
	• Panel members indicate (dis)agreement to all items including the additionally suggested aspects
Round 3	• Panel members indicate (dis)agreement on the agreed-upon list of the previous rounds
	• In case of disagreement, panel members specify changes required to agree with the list of aspects

Based on the differences between animal and human research [44], the present study will focus on WBV studies with human subjects only. A separate Delphi study will be conducted to initiate reporting guidelines for animal and cell culture studies.

The rounds were conducted consecutively starting in February 2019 and the conclusion of the last round in May 2019. The panel members were asked to complete each questionnaire within three weeks. A weekly reminder to participate in the current round was sent to the experts who did not complete the questionnaire yet. In the first round, potential panel experts who are members of the WAVEX received an additional reminder to participate from the board of the association after two weeks. All questionnaires were created with the online survey software Qualtrics (Version 02/2019-05/2019 of Qualtrics; Copyright © 2019 Qualtrics; Provo, UT, USA [45]) and distributed via Qualtrics by e-mail. This software additionally ensured anonymous data collection throughout the whole study process.

The responses to the individual questionnaires were treated confidentially and the study was approved by the Ethical Committee Psychology (ECP) affiliated to the University of Groningen, the Netherlands on 20/12/2018 (ref. no.; 18211-O).

### Criteria regarding consensus achievement

Consensus about the aspects which should be included in reporting guidelines was is defined as an agreement (*yes*, *of importance to report*) of at least 70% [46,47] while the disagreement rate [48,49] (*no*, *not important to report*) is below 20% prior to the start of the study (see Table 2). An agreement of 20% or below and a disagreement of 20% or above by the expert panel is regarded as judging the item irrelevant. In case items were neither agreed nor disagreed upon (below 70% of agreement and below 20% of disagreement or agreement above 70% and disagreement above 20%) or fall directly on the set criteria (e.g., an agreement of exactly 20%), the items are named as ‘optional items’ in the final list of aspects of the present study, which may be valuable in certain studies, as automatic exclusion may not be justified [50]. These criteria were set by the executive group after consulting the literature of Delphi studies and were judged to reasonably indicate consensus. Yet, based on the lack of agreement about the definition of consensus [41,46], these values are to be seen as indicative of agreement and not as an absolute judgement.

**Table 2:** Overview of consensus criteria specifying the agreement-rates and corresponding disagreement-rates.

Consensus of	Agreement-Rate	Disagreement-Rate
-Important to report	≥70%	≤20%
-Not important to report	≤20%	≥20%
Optional item	>70%	>20%
	20% - 70%	≤20%

The results are analyzed quantitatively with: (1) Krippendorff’s alpha to estimate overall agreement per round [51–53]; (2) the McNemar χ^2^-test to estimate stability of ratings between round 1 and 2 [46,54]; (3) and Cohen’s Kappa to estimate the agreement between round 1 and 2 per item [55,56]. These estimates can be found in the supporting information (S3 File) since violations of the assumptions of these estimates may reduce their validity.

### Participants and recruitment

Suitable panel members with expertise of WBV from any scientific discipline making use of WBV in human studies are essential to the present study. The inclusion criteria were designed to ensure the high expertise of possible panel members. Thus, the inclusion criteria were the following: (1) potential panel members must be willing to take part in the study and (2) have at least two English, scientific (peer-reviewed) publications utilizing WBV with human participants. Exclusion criteria were: (1) one English scientific (peer-reviewed) publication utilizing WBV with human participants and (2) if two or more scientific (peer-reviewed) publications were published but in a language other than English (i.e., only no or one English peer-reviewed publications) possible experts were excluded. Experts were identified through membership in the WAVEX (https://internationalwavexmeeting.wordpress.com/), publications determined via the scientific databases PubMed, PsycInfo, and Web of Science, and by recommendations of members of WAVEX. Based on this a list of potential experts was compiled and invitations to participate in the study were sent to them. Additionally, the experts who received an invitation had the opportunity to recommend further experts which could take part in the study.

In total 136 experts were contacted. Fifty-six of these opened and filled in parts of the first questionnaire (41.2% response rate). Fifty-one of these 56 participants completed the questionnaire (51/56 = 91.1% completion rate). The expert panel consisted of the 51 participants who completed the first questionnaire and only these experts were contacted in the subsequent round. In the second round, 40 panel members completed the survey (78% response rate). Only the experts who completed round 2 were invited to the third round. Thirty-seven of these 40 experts completed round 3 (92.5% response rate).

### Materials and procedure

**Fig 1:**
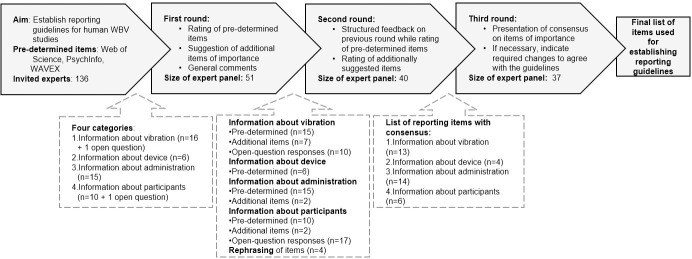
Flowchart of the Delphi study building towards reporting guidelines in human WBV studies. ‘n’ refers to the number of items in each category.

An overview of the procedure of the present study is given in Fig 1 and the full instructions and questionnaires of all three rounds can be found in the supporting information (S2 File).

#### Round 1

The first round had two aims: (1) to receive an initial rating whether the pre-determined items should be included in guidelines concerning WBV studies with humans and (2) to collect suggestions of additional aspects the experts see as relevant. In the beginning of this round, the expert panel members received information about the aim of the study and, after giving informed consent, were asked to answer several general demographic questions. In addition to age and gender, participants also indicated their highest completed education, academic background and position, years of experience with research and clinical work, the current distribution of their time (research/clinical/other), years of experience with WBV in research and clinical settings, and number of published studies utilizing WBV.

After completing the short demographic questionnaire, the experts were presented with the pre-determined items of importance. These 48 pre-determined items were separated into four categories: *information about the* (1) *vibration*, (2) *device*, (3) *administration*, and (4) *participants*. The experts were asked to indicate whether they agree (‘yes’), do not agree (‘no’), or are neutral (‘neutral/ don’t know’) about the importance of each specific item when reporting human WBV studies. After each block, the experts had the opportunity to suggest additional items and add questions or comments.

Two open questions were included (item 6 and 36). If experts agreed with item 6 (*reporting how vibration parameters are measured*), they were asked to suggest how the measurements should be completed. Similarly, if experts agreed with item 36 (*reporting of participants subjective experiences during WBV*), they were asked to list the, in their opinion, relevant experiences.

#### Round 2

The aim of the second round was twofold. First, the experts were asked to indicate their judgement about the items of the first round, while taking the first rounds ratings of the panel into account. Hence, the experts re-evaluate their initial responses to the items in light of their fellow experts’ opinion. This process is thought to be crucial to establish consensus, as convergence of the individual opinions occurs [57]. Second, the additionally suggested items and the results of the two open questions of the first round were presented to the experts. Similar to the pre-determined items, the experts’ judgement on whether to include these items in reports of human WBV studies was inquired.

#### Round 3

Similar to round 2, the items with consensus (agreement of at least 70% while the disagreement rate is below 20%) regarding their importance to report, along with their ratings of the second round, were presented.

The aim of the third round was to seek the experts’ agreement on the final list of items. The panel members were asked to indicate agreement (yes/no) with the whole agreed-upon list based on the previous rounds (see Fig 1). It was pointed out to the experts that not all items may generally apply to each WBV study. However, items may still be relevant for particular studies. In case of disagreement, the experts were asked to specify the reason for disagreement, i.e., which aspect they disagree with.

## Results

### Round 1

#### Sample characteristics

Table 3 gives an overview of the demographic characteristics of the 51 expert panel members. 41.2% of the experts were female and the experts’ age was distributed roughly equally across age groups with a mean of M = 46 years (SD = 10.4 years) ranging from 27 to 68 years. Most experts obtained a PhD (n = 30) and fewer a master’s degree (n = 4) or a professorship (n = 17) and had an average of 18 years of research experience and 11 years of clinical experience (Table 3). The panel experts were located in 17 different countries. Various academic backgrounds were reported, including medicine, engineering, physiotherapy, psychology, and movement sciences. The expert panel reported an average of 11 publications utilizing WBV with humans ranging from 2 to 40. Additionally, 37 experts reported clinical experience with WBV.

**Table 3:** Demographic characteristics of the expert panel.

	Round 1	Round 2	Round 3
N	51	40	37
Age (in years, *M ± SD*) ^a^	45.8±10.4	47.1±10.3	46.6±10.3
Gender (female/male)	21/30	15/25	13/24
Geographic location			
Brazil	16	10	9
France	3	2	2
Germany	6	6	6
Italy	5	5	5
Spain	3	2	2
The Netherlands	4	3	3
USA	4	4	3
Other	10 ^b^	8^c^	7^d^
Academic background			
Engineering	10	8	8
Exercise physiology	3	3	2
Medicine	9	7	7
Movement/Health/Sport sciences	11	10	9
Physical therapy	16	10	9
Psychology	2	2	2
Academic degree/position (MSc/ PhD/ Prof)	4/30/17	1/23/16	1/21/15
Research work experience (in years, *M ± SD*)	18.1±10.7	19.7±10.7	19.4±10.6
Clinical work experience (in years, *M ± SD*)	11.5±10.1	11.2±9.9	11.6±9.9
Distribution of work time (in %, *M ± SD*)			
Clinical work	17.9±24.4	17.3±23.3	17.7±23.6
Research	48.0±23.5	48.1±22.8	49.3±23.1
Other	34.1±24.9	34.5±24.5	32.9±23.8
Number of publications utilizing WBV (*M ± SD*)	11.4±10.2	12.02±10.9	11.6±11.2
Clinical experience with the use of WBV (yes/no)	37/14	31/9	30/7

^a^ two non-responses; one expert each from Australia ^b, c, d^, Belgium ^b, c^, Canada ^b, c, d^, Colombia ^b, c, d^, Finland ^b, c, d^, New Zealand ^b, c, d^, South Africa ^b, c, d^, Switzerland ^b^, United Arab Emirates ^b^, United Kingdom ^b, c, d^.

#### Agreement on pre-determined items

The ratings of the pre-determined list can be found in Table 4. For the first category *information about the vibration* 14 out of 16 items had an agreement rating of at least 70% (items 1–14). For item 15 (*report the accuracy of vibration parameters*), 68.6% of the experts were in favor of reporting the item. The remaining experts responded ‘neutral/do not know’ (23.5%) rather than ‘no’ (7.8%). For item 16 (*which acceleration (in RMS) occurs*), 37.3% of the experts were in favor of reporting. More than 20% of the experts voted against reporting this item, while 39.2% did not make a definite decision (‘neutral’).

**Table 4:** Pre-determined items of importance when reporting human WBV studies and their importance rating of round 1 and 2.

	Round 1				Round 2			
Item Number	When considering human studies using whole-body vibration is it important to report. . .	Rating Yes/Neutral/No	Agreement (% of yes)	Disagreement (% of no)	When considering human studies using whole-body vibration is it important to report. . .	Rating Yes/Neutral/No	Agreement (% of yes)	Disagreement (% of no)
	*Information about vibration*				*Information about vibration*			
1	…which frequency (Hz) is used?	51/0/0	**100.0**	0.0	. . .which frequency (Hz) is used?	39/0/1	**97.5**	2.5
2	…whether the frequency (Hz) is constant or variable?	50/1/0	**98.0**	0.0	. . .whether the frequency (Hz) is constant or variable?	37/0/3	**92.5**	7.5
3	…the type of vibration (i.e. synchronous, side-alternating, other)?	49/0/2	**96.1**	3.6	…the type of vibration (i.e. synchronous, side-alternating, other)?	40/0/0	**100.0**	0.0
4	…the units of the vibration parameters?	48/1/2	**94.1**	3.9	. . .the units of the vibration parameters?	40/0/0	**100.0**	0.0
5	…the amplitude of the vibration (in mm)?	48/2/1	**94.1**	2.0	. . .the amplitude of the vibration (in mm)?	40/0/0	**100.0**	0.0
6*	…how the vibration parameters are measured, if own settings are used?	44/5/2	**86.3**	3.9	. . .how the vibration parameters are measured	34/3/3	**85.0**	7.5
7	…the definitions/explanations of the vibration parameters?	41/3/7	**80.4**	13.7	. . .the definitions/explanations of the vibration parameters?	31/6/3	**77.5**	7.5
8	…which peak-acceleration (in multiples of g) occurs?	41/7/3	**80.4**	5.9	. . .which peak-acceleration (in multiples of g) occurs?	32/5/3	**80.0**	7.5
9	…the position of each foot on the base of the vibration platform to calculate PDD or amplitude for side-alternating vibration?	41/8/2	**80.4**	3.9	. . .the position of each foot on the base of the vibration platform to calculate PDD or amplitude for side-alternating vibration?	37/2/1	**92.5**	2.5
10*	…the position of the platform where PDD or amplitude is measured?	41/9/1	**80.4**	2.0	*Incorporated in item 14*			
11	…whether manufacturer settings or own settings are used?	40/5/6	**78.4**	11.8	. . .whether manufacturer settings or own settings are used?	34/2/4	**87.5**	5.0
12	…whether the vibration is immediately full or increases slowly?	40/6/5	**78.4**	9.8	. . .whether the vibration is immediately full or increases slowly?	34/3/3	**85.0**	10.0
13	…which peak-to-peak displacement (PDD) of the vibration occurs?	40/7/4	**78.4**	7.8	. . .which peak-to-peak displacement (PDD) of the vibration occurs?	33/5/2	**85.0**	7.5
14*	…where on the platform the vibration parameters are measured, if own settings are used?	38/10/3	**74.5**	5.9	. . .where on the platform the vibration parameters are measured?	35/3/2	**82.5**	5.0
15	…what the accuracy of vibration parameters is?	35/12/4	68.6	7.8	. . .what the accuracy of vibration parameters is?	22/13/5	55.0	12.5
16*	…which acceleration (in RMS) occurs?	19/20/12	37.3	*23*.*5*	. . .which acceleration occurs based on a pilot study or relevant literature?	13/18/9	32.5	*22*.*5*
	Additional suggestions	27/24 ^a^	52.9	47.1				
	*Information about device*				*Information about device*			
17	…whether the device vibrates horizontal, vertical, side-alternating, waveform changing, or other?	50/0/1	**98.0**	2.9	. . .whether the device vibrates horizontal, vertical, side-alternating, waveform changing, or other?	40/0/0	**100.0**	0.0
18	…whether changes are made to the device (e.g. mounting a chair on it)?	49/2/0	**96.1**	0.0	. . .whether changes are made to the device (e.g. mounting a chair on it)?	39/1/0	**97.5**	0.0
19	…the manufacturer, device specifications, and production type?	48/1/2	**94.1**	3.9	. . .the manufacturer, device specifications, and production type?	39/1/0	**97.5**	0.0
20	…whether a handrail is available?	42/4/5	**82.4**	9.8	. . .whether a handrail is available?	34/3/3	**85.0**	7.5
21	…how the energy is generated (e.g. direct mechanical transmission or electromagnetic transmission) as this can have an effect on the performance of the device?	28/19/4	54.9	7.8	. . .how the energy is generated (e.g. direct mechanical transmission or electromagnetic transmission) as this can influence the performance of the device?	20/12/8	50.0	*20*.*0*
22	…the size of the vibration platform?	25/14/12	49.0	*23*.*5*	. . .the size of the vibration platform?	17/10/13	42.5	*32*.*5*
	Additional suggestions	9/42 ^a^	17.6	82.4				
	*Information about administration*			*Information about administration*		
23	…which posture or body position the participants take on during the vibration (e.g. sitting, standing, squatting)?	51/0/0	**100.0**	0.0	. . .which posture or body position the participants take on during the vibration (e.g. sitting, standing, squatting)?	40/0/0	**100.0**	0.0
24	…whether the position/posture changes during the WBV (static versus dynamic exercise)?	51/0/0	**100.0**	0.0	. . .whether the position/posture changes during the WBV (static versus dynamic exercise)?	40/0/0	**100.0**	0.0
25	…the number of sessions where WBV was utilized?	51/0/0	**100.0**	0.0	. . .the number of sessions where WBV was utilized?	40/0/0	**100.0**	0.0
26	…the resting time between sessions of WBV?	51/0/0	**100.0**	0.0	. . .the resting time between sessions of WBV?	40/0/0	**100.0**	0.0
27	…the number of exposures to WBV within one session?	50/1/0	**98.0**	0.0	. . .the number of exposures to WBV within one session?	39/1/0	**97.5**	0.0
28	…where the feet of the participants are placed?	49/2/0	**96.1**	0.0	. . .where the feet of the participants are placed?	30/1/0	**97.5**	0.0
29	…the total exposure time to WBV across all sessions?	48/1/2	**94.1**	3.9	. . .the total exposure time to WBV across all sessions?	39/0/1	**97.5**	2.5
30	…where the hands of the participants are placed?	47/3/1	**92.2**	2.0	. . .where the hands of the participants are placed?	37/1/2	**92.5**	5.0
31	…which exact tools and aids were used during the WBV (e.g. type and size of dumbbells)?	46/4/1	**90.2**	2.0	. . .which exact tools and aids were used during the WBV (e.g. type and size of dumbbells)?	36/3/1	**90.0**	2.5
32	…the on vs. off-times of vibration (e.g. pauses and how long) within one session?	45/5/1	**88.2**	2.0	. . .the on vs. off-times of vibration (e.g. pauses and how long) within one session?	37/1/2	**92.5**	5.0
33	…whether an examiner was present to supervise the WBV administration?	43/3/5	**84.3**	9.8	. . .whether an examiner was present to supervise the WBV administration?	34/2/4	**85.0**	10.0
34*	…whether the cognitive/physical outcome measures are assessed during or after the WBV?	41/5/5	**80.4**	9.8	. . .whether the outcome measures (cognitive/physical) are assessed during or after the WBV?	35/2/3	**87.5**	7.5
35	…possible follow-ups to determine possible lasting effects?	41/7/3	**80.4**	5.9	. . .possible follow-ups to determine possible lasting effects?	34/2/4	**85.0**	10.0
36	…how resonance (skidding of feet) is prevented (e.g. front-foot vs. mid-foot stance)?	39/9/3	**76.5**	5.9	. . .how resonance (skidding of feet) is prevented (e.g. front-foot vs. mid-foot stance)?	27/8/5	67.5	12.5
37	…the location of the intervention (e.g. hospital or gym)?	30/8/13	58.8	*25*.*5*	. . .the location of the intervention (e.g. hospital or gym)?	28/7/5	**70.0**	12.5
38	…the conditions of the test room (e.g. light and temperature)?	24/10/17	47.1	*33*.*3*	. . .the conditions of the test room (e.g. light and temperature)?	20/6/14	50.0	*35*.*0*
	Additional suggestions	10/41 ^a^	19.6	80.4				
	*Information about participants*				*Information about participants*			
39	…subjective experiences of participants before, during, or after the WBV (e.g. dizziness, pain, postural instability, muscle fatigue)?	46/4/1	**90.2**	2.0	. . .subjective experiences of participants before, during, or after the WBV (e.g. dizziness, pain, postural instability, muscle fatigue)?	37/1/2	**92.5**	5.0
40*	…the participants' BMI (height, weight)?	45/2/4	**88.2**	7.8	. . .the participants' height and weight?	39/0/1	**97.5**	2.5
41	…the participants' footwear (shoes, socks, barefoot) during WBV with a detailed description?	45/4/2	**88.2**	3.9	. . .the participants' footwear (shoes, socks, barefoot) during WBV with a detailed description?	38/1/1	**95.0**	2.5
42	…if, how, and for how long participants prepared for the WBV (e.g. stretching, muscles, warm up)?	43/6/2	**84.3**	3.9	. . .if, how, and for how long participants prepared for the WBV (e.g. stretching, muscles, warm up)?	36/3/1	**90.0**	2.5
43	…the participants' fitness and activity levels?	43/7/1	**84.3**	2.0	. . .the participants' fitness and activity levels?	37/2/1	**92.5**	2.5
44	…how it is assured that neck and head are not negatively affected by the vibration?	33/10/8	64.7	15.7	. . .how it is assured that neck and head are not negatively affected by the vibration?	20/10/10	50.0	*25*.*0*
45	…the participants' clothing during the WBV?	24/12/15	47.1	*29*.*4*	. . .the participants' clothing during the WBV?	15/11/14	37.5	*25*.*0*
46	…skin and muscle perfusion during the WBV?	16/15/20	31.4	*39*.*2*	. . .skin and muscle perfusion during the WBV?	5/15/20	12.5	*50*.*0*
47	…whether possible glasses were disturbing during the vibration?	14/20/17	27.5	*33*.*3*	. . .whether possible glasses were disturbing during the vibration?	6/12/22	15.0	*55*.*0*
48	…whether the participants wore glasses during WBV?	13/18/20	25.5	*39*.*9*	. . .whether the participants wore glasses during WBV?	6/9/25	15.0	*62*.*5*
	Additional suggestions	10/41 ^a^	19.6	80.4				

Questions marked ^a^ had only yes/no answer options

* indicates a rephrasing of the item for the second round; **bold** indicates agreement of 70% or higher and no disagreement of 20% or higher; *italic* indicates a disagreement of 20% or higher; the dotted box depicts the incorporation of item 10 into item 14.

For the items referring to the *information about the device*, four of six items were agreed upon (items 17–20). The two items of no-consensus; item 21 (*how the energy is generated (e*.*g*. *direct mechanical transmission or electromagnetic transmission) as this can influence the performance of the device*) and item 22 (*how the energy is generated the size of the vibration platform*) had a higher rating of ‘neutral/do not know’ than favoring against reporting it.

*Information about the administration* items were generally agreed upon for 14 of the 16 items (item 23–36 with an agreement of 70% or higher). For item 37 (*reporting the location of the intervention*) and 38 (*reporting the conditions of the test room*) most experts not in favor voted against reporting these items.

Five of the ten items concerning *information about the participants* were agreed upon (items 39–43). For item 44 (*how it is assured that neck and head are not negatively affected by the vibration*) and 47 (*whether possible glasses were disturbing during the vibration*), most experts not in favor of reporting responded with ‘neutral/do not know’. Most of the remaining experts voted against reporting item 45 (*the participants' clothing during the WBV*), 46 (*skin and muscle perfusion during the WBV*), and 48 (*whether the participants wore glasses during WBV*).

All answers regarding the two open questions can be found in the available dataset. For item 6 (*how should vibration parameters be measured*), the use of an accelerometer was suggested 24 times. Other suggestions were brought forward three times or less. These answers were rated for their importance in the second round of the study (see Table 5).

**Table 5:** Round 2 ratings of the additionally suggested items of round 1.

Items [number of times suggested]	Rating Yes/Neutral/No	Agreement (% of yes)	Disagreement (% of no)
*Additional items*: *Information about vibration*			
…always measure vibration parameters, even if manufacturer settings are used? [1x]	26/3/11	65.0	*27*.*5*
…the vertical and horizontal accelerations? [1x]	25/8/7	62.5	17.5
…how the subject's body mass affects the vibration parameters (compare measure with and without subject on the platform)? [2x]	24/5/11	60.0	*27*.*5*
…report whether manufacturer settings are used but additionally report own vibration parameter measurements? [2x]	23/11/6	57.5	15.0
…the intensity of the exposure (in m/s^2 RMS)? [1x]	19/9/12	47.5	*30*.*0*
…if manufacturer settings are used, is it sufficient to report these? [1x]	19/6/15	47.5	*37*.*5*
…sEMGs (surface electromyography) to evaluate and record the electrical signals produced by muscle activity [2x]	12/10/18	30.0	*45*.*0*
*How should vibration parameters be measured*?			
…with frequency and amplitude [4x]	37/2/1	**92.5**	2.5
…with the aid of accelerometers [24x]	31/3/6	**77.5**	15.0
*If yes*: *how should accelerometers be used*?	*Count*	*%*	
3D-accelerometer for vertical and horizontal acceleration	10	32.5	
Accelerometer at vibration platform only	4	12.9	
Accelerometer on participant only (e.g. at joints)	0	0.0	
Accelerometer at vibration platform and on participant	14	45.2	
It does not matter how they are measured, as long as they are measured	1	3.2	
Other	2	6.5	
…with frequency, amplitude, and peak-to-peak displacement [1x]	28/8/4	**70.0**	10.0
…with frequency and amplitude over time [1x]	19/13/8	47.5	*20*.*0*
Vibration parameters should be measured to the individual case [1x]	16/12/12	40.0	*30*.*0*
…with frequency and amplitude including RMS (in multiples of g) [1x]	15/13/12	37.5	*30*.*0*
…with peak-to-peak amplitude rather than RMS [1x]	14/17/9	35.0	*22*.*5*
It is not important how they are measured but that they are reported [1x]	12/8/20	30.0	*50*.*0*
…with laser Distance Sensor [2x]	6/19/15	15.0	*37*.*5*
…with gyroscope [1x]	4/19/17	10.0	*42*.*5*
*Additional items*: *Information about administration*			
…to report whether only parts of the subjects' body are subjected to vibration (e.g. only the feet)? [2x]	33/4/3	**82.5**	7.5
…to explain the decision which parts (e.g. only feet) of the participants are subjected to vibration and why [1x]	28/4/8	**70.0**	*20*.*0*
*Additional items*: *Information about participants*			
…training history [3x]	33/4/3	**82.5**	10.0
…history of injuries [1x]	27/11/5	60.0	12.5
*Which subjective experiences should be reported*?			
Side effects/adverse effects	40/0/0	**100.0**	0.0
Pain (location, intensity, type)	36/3/1	**90.0**	2.5
Dizziness	35/3/2	**87.5**	5.0
(Dis)comfort	33/4/3	**82.5**	7.5
Fatigue/exhaustion/tiredness	32/5/3	**80.0**	7.5
Tingling/itching/burning sensations	32/7/1	**80.0**	2.5
Perceived exertion/effort (e.g. with Borg RPE scale)	31/6/3	**77.5**	7.5
Muscle soreness/weakness	30/7/3	**75.0**	7.5
Headache	30/7/3	**75.0**	7.5
Loss of balance	29/7/4	**72.5**	10.0
Vertigo	26/8/6	65.0	15.0
Subjective experience depends on the aim of the study and cannot be generalized	25/9/6	62.5	15.0
Nausea	24/9/7	60.0	17.5
Redness	23/10/7	57.5	17.5
Enjoyment	19/10/11	47.5	*27*.*5*
Mood	18/12/10	45.0	*25*.*0*

**Bold** indicates agreement of 70% or higher and no disagreement of 20% or higher; *italic* indicates a disagreement of 20% or higher.

Six responses to item 39 (*which subjective experiences of WBV should be reported*) were suggested at least four times. These items are (1) level of pain, (2) dizziness, (3) fatigue, exhaustion, and/ or tiredness, (4) tingling, itching, and/or burning sensations, (5) perceived effort or exertion, (6) experiences of (dis)comfort. Other suggestions were brought forward three times or less and are included in the available dataset.

#### Suggested items

A list of additionally suggested items and comments for each of the four categories are included in the dataset. The first part of the questionnaire *regarding information about the vibration* elicited several comments and additional items.

First, it was suggested twice that surface electromyography (sEMG) should be included to evaluate and record the electrical signals produced by muscle activity. Second, it was suggested twice that the way the participants’ body mass might influence the vibration parameters should be measured. It has further been suggested that this could be done by comparing the vibration parameters of the empty vibration device and while a participant is using it. Third, it was suggested once that the acceleration in ms^-2^ RMS should be reported. Fourth, it was suggested that accelerations should be measured vertically and horizontally.

For *information about the vibration*, it was suggested twice that regardless of using own or manufacturer settings (item 11), the vibration parameters should always be measured, as they can be faulty. Therefore, several questions were added to the questionnaire of round 2. First, whether it should be reported if manufacturer settings are used but additionally report own vibration parameter measurement; second, whether it is sufficient to report manufacturer settings without additional measurements; and third, whether vibration parameters should always be measured regardless whether manufacturer settings are used or not.

For the second category *information about the device* some additional items were suggested. Yet, they were judged to be unrelated to this category (see dataset for the comments) and thus no additional questions from these comments were added to the questionnaire of round 2.

For *information about the administration*, two additional items were suggested. First, reporting whether parts of the subjects are exposed to vibration in contrast to whole body vibration (e.g., only the feet) was suggested twice. Second, it was suggested once to report the decision which parts of the subjects are exposed to vibration and why.

For *information about the participants* two additional items were suggested. First, it was commented three times that the training history of the subjects should be reported. Second, it was suggested once to report the subjects’ history of injuries.

All comments and additional suggestions are included in the dataset.

### Round 2

#### Sample characteristics

See Table 3 for an overview of the demographic characteristics of the 40 panel members that completed round 2.

#### Integration of comments from round 1

In round 2, three of the items of the first round were rephrased based on comments from the experts. Item 8 (*report which acceleration occurs*) was rephrased to *which acceleration occurs based on pilot study or relevant literature*. Item 14 *(where on the platform the vibration parameters are measured*, *if own settings are used*) was rephrased to *where on the platform the vibration parameters are measured*, since it was commented by the expert panel that the vibration parameters have to be assessed since the information of the manufacturers may be inaccurate. Item 10 (*the position on the platform where peak-to-peak displacement (PPD) or amplitude is measured*) was rephrased due to comments of the expert that the phrasing was not optimal. It was incorporated into item 14 since PDD and amplitude are vibration parameters.

#### Agreement ratings

The results of the judgement on the pre-determined items which were presented along with the ratings of the first round can be found in Table 4.

For items previously judged as important, agreement rates mostly increased in all four categories (see Table 4). For the first category *information about the vibration* the same items as in the first round had an agreement of at least 70% and no disagreement over 20% (items 1–14 excluding item 10). For all, except four items (1, 2, 6, 7), the agreement rate increased. Similarly to the first category, the second category *information about the device* also showed similar ratings in round 2 as in round 1. Again, the items previously judged as important to report, received higher agreement rates in the second round (items 17–20). *Information about the administration* items were agreed upon for the same items (items 23–35 with an agreement of 70% or higher) of the first round. As depicted in Table 4, ratings of item 36 fell below the 70% threshold and the ratings of item 37 increased. The same items regarding *information about the participants* were agreed upon (items 39–43) in round 2 and the agreement-rate increased for all.

The ratings of the additional suggested items and the responses of the open questions are presented in Table 5. For the additional items concerning *information about vibration*, no item was agreed-upon in terms of necessity of reporting. In the first round, the question *how should vibration parameters be measured*? was answered by the experts. These answers were rated in the second round (see Table 5). An agreement was established for the use of accelerometers (77.5%), with frequency and amplitude (92.5%), and frequency, amplitude, and peak-to-peak displacement (70%). Since various usages of accelerometers were suggested in the first round, the experts were additionally asked to rate how the accelerometer should be used, if agreed to it. The majority voted for the use of 3D-accelerometers on both participant and platform and horizontally and vertically (see Table 5).

Two additional items were suggested for the *information about the administration* (see Table 5). Both reached an agreement-rate of 70% or higher (82.5% and 70%). However, one of these items also reached a disagreement-rate of 20% (see Table 5).

For the category *information about the participants* two suggestions were made in the first round. The first suggestion was agreed-upon, while the second was not (see Table 5). Supplementary to the additional suggestions, the answers of the open question of which subjective experience should be reported were rated. Ten items were agreed upon and two were disagreed upon (see Table 6).

**Table 6:** Final list of aspects of human WBV studies after round 3 of this Delphi study.

Category	Items
*Information about vibration*	1. The type of vibration (i.e., synchronous, side-alternating, other)
	2. The units of the vibration parameters
	3. The amplitude of the vibration (in mm)
	4. Which frequency (Hz) is used
	5. The position of each foot on the base of the vibration platform to calculate PDD or amplitude for side-alternating vibration
	6. Whether the frequency (Hz) is constant or variable
	7. Whether manufacturer settings or own settings are used
	8. How the vibration parameters are measured
	9. Whether the vibration is immediately full or increases slowly
	10. Which peak-to-peak displacement (PDD) of the vibration occurs
	11. Where on the platform the vibration parameters are measured
	12. Which peak-acceleration (in multiples of g) occurs
	13. The definitions/explanations of the vibration parameters (see [1,6,23,32])
	14. Vibration parameters should be measured with
	• frequency and amplitude, *or*
	• frequency, amplitude, and peak-to-peak displacement, *or*
	• the aid of 3D-accelerometers
	a. with 3D-accelerometer at vibration platform and on participant, *and/or* b. 3D-accelerometer for vertical and horizontal acceleration
*Information about device*	15. Whether the device vibrates horizontal, vertical, side-alternating, waveform changing, or other)
	16. Whether changes are made to the device (e.g., mounting a chair on it)
	17. The manufacturer, device specifications, and production type
	18. Whether a handrail is available
*Information about administration*	19. Which posture or body position the participants take on during the vibration (e.g., sitting, standing, squatting)
	20. Whether the position/posture changes during the WBV (static versus dynamic exercise)
	21. The number of sessions where WBV was utilized
	22. The resting time between sessions of WBV
	23. The number of exposures to WBV within one session
	24. Where the feet of the participants are placed
	25. The total exposure time to WBV across all sessions
	26. Where the hands of the participants are placed
	27. The on vs. off-times of vibration (e.g., pauses and how long) within one session
	28. Which exact tools and aids were used during the WBV (e.g. type and size of dumbbells)
	29. Whether the outcome measures (cognitive/physical) are assessed during or after the WBV
	30. Whether an examiner was present to supervise the WBV administration
	31. Possible follow-ups to determine possible lasting effects
	32. To report whether only parts of the subjects' body are subjected to vibration (e.g., only the feet)
	33. The location of the intervention (e.g., hospital or gym)
	34. To explain the decision which parts (e.g., only feet) of the participants are subjected to vibration and why
*Information about participants*	35. The participants' height and weight
	36. The participants' footwear (shoes, socks, barefoot) during WBV with a detailed description
	37. Subjective experiences of participants before, during, or after the WBV
	• Side effects/adverse effects
	• Pain
	• Dizziness
	• (Dis)comfort
	• Fatigue/exhaustion/tiredness
	• Tingling/itching/burning sensations
	• Perceived exertion/effort (e.g., with Borg RPE scale)
	• Muscle soreness/weakness
	• Headache
	• Loss of balance
	38. The participants' fitness and activity levels
	39. If, how, and for how long participants prepared for the WBV (e.g., stretching, muscle warm up)
	40. Training history
*Optional items*	1. The vertical and horizontal accelerations
	2. Which acceleration occurs based on a pilot study or relevant literature
	3. Report whether manufacturer settings are used but additionally report own vibration parameter measurements
	4. What the accuracy of vibration parameters is
	5. How resonance (skidding of feet) is prevented (e.g. front-foot vs. mid-foot stance)
	6. How the subject's body mass affects the vibration parameters (compare measure with and without subject on the platform)
	7. The intensity of the exposure (in m/s^2 RMS)
	8. Always measure vibration parameters, even if manufacturer settings are used
	9. If manufacturer settings are used, it is sufficient to report these
	10. sEMGs (surface electromyography) to evaluate and record the electrical signals produced by muscle activity
	11. Measurements of vibration parameters:
	a. With frequency and amplitude over time
	b. Vibration parameters should be measured to the individual case
	c. With frequency and amplitude including RMS (in multiples of g)
	d. With peak-to-peak amplitude rather than RMS
	e. It is not important how they are measured but that they are reported
	12. How energy is generated (e.g. direct mechanical transmission or electromagnetic transmission) as this may influence the performance of the device
	13. The size of the vibration platform
	14. The conditions of the test room (e.g. light and temperature)
	15. Explain the decision which parts (e.g. only feet) of the participants are subjected to vibration and why
	16. Other subjective experiences
	• Vertigo
	• Nausea
	• Redness
	• Enjoyment
	• Mood
	17. History of injuries
	18. How it is assured that neck and head are not negatively affected by the vibration
	19. The participants’ clothing during the WBV

### Round 3

#### Sample characteristics

See Table 3 for an overview of the demographic characteristics of the 37 panel members that completed round 3.

#### Final agreements

The final, agreed-upon, list can be found in Table 6. Thirty-five of the 37 experts comprising the expert panel in the last round, agreed with this final list (agreement of 94.6%). One of the experts disagreed with the *subjective experiences* of the list, without giving further explanations. The second disagreement concerned the statement of ‘*how vibration parameters should be measured’* with the explanations that these recommendations are mutually exclusive, and it needs to be clarified whether 1D- or 3D-accelerometers should be utilized. The statements of the two disagreeing experts can be found in the available dataset.

## Discussion

The aim of the present study was to lay an empirical foundation for the first steps in the development of reporting guidelines for all scientific disciplines applying WBV as a training or treatment method to human subjects. This was based on the recommendations by the EQUATOR Network [36] with a three-round Delphi method. As elaborated in the introduction, a need for updating and extending the recommendations by Rauch et al. [1] was identified on the grounds of creating the guidelines in a systematic fashion and the still persisting incomplete reporting and misunderstandings of terminology. The combination of these aspects may hamper the comparability of studies, interferes with their replicability, and ultimately prevents the achievement of pivotal insights into the effects of WBV. Therefore, the knowledge and opinions of international experts working with WBV was collected and integrated.

The Delphi method is widely used due to its advantages. First, it can be applied to groups of varying size [58]. Second, it is thought to minimize issues of collective decision making, such as reciprocal influence and lack of anonymity [37] and, thus, prevents the imposition of opinions from dominant individuals [59]. Third, with the Delphi method, the opinion of a large number of individuals with diverse geographical and professional backgrounds is collectible. Fourth, anonymity allows the participants to opinionate unpopular aspects and change their responses [60]. Therefore, the utilization of the Delphi method appears appropriate to lay the foundation for the reporting guidelines and as the methods of the present study.

Throughout this Delphi study, a “yes”/“no”/“neutral/don’t know” rating system is used. The experts are asked to consider whether one specific item should be reported in a publication of a WBV study or not. Additionally, the experts are instructed to consider the whole field of WBV, as opposed to personal studies, since the guidelines should be relevant for all applications of WVB. Thus, another rating system was judged to reduce the explanatory power of the results due to a missing indication about the reasoning why an item was judged, for example as “very important” compared to “important”.

In the next step, the executive group will use the generated list of the Delphi study to develop the reporting guidelines, according to the EQUATOR Network [36] recommendations. Issues that will be considered are (1) modifications of items (e.g., rephrasing), (2) adding further items deemed as important (e.g., define the boundaries of low and high frequency), (3) discussing the comments of disagreeing experts and potential consequences for the guidelines, (4) decide about the inclusion/exclusion of the optional items, and (5) the publication and dissemination strategy (e.g., register at EQUATOR Network).

The three rounds of the present study ensured that the expert panel was able to refine their judgements about the pre-determined items, suggest additional items, and rate all items repeatedly. A large consensus on the final list of aspects was established according to this Delphi study (94.6%). The results show high levels of consensus for various items, as judged by an agreement rate of 70% while the disagreement rate was below 20%. The items which attained consensus were included in the final list (see Table 6).

Even though most items reached either an agreement or disagreement, some items were left without consensus after the three rounds (i.e., no agreement of 70% or higher and no disagreement of 20% or higher). We propose that these optional items could be individually considered for reporting as they can be valuable for particular studies. There are 19 optional items (see Tables 3 and 4 for ratings). These items were disproportionally often rated as *neutral* compared to items of consensus (see Tables 4 and 5). It can be speculated that either unfamiliarity with the item or ambiguously posed questioning of the item were reason of reaching no consensus. Since items were rephrased throughout the three rounds, ensuring proper and unambiguous phrasing, possible unfamiliarity with items is conceivable. Thus, considering the optional items for future WBV studies and reporting is sensible depending on the study. The final decision about these items will be completed by the executive group and reported on in the new guidelines.

The identified items of the final list largely correspond to the advice given by previous publications concerning the terminology and vibration parameters [6,32] as well as the reporting recommendations of WBV studies [1]. The aspects discussed by Lorenzen et al. [32] (i.e., magnitude of vibration), which are commonly misused or misunderstood, are in line with the final list of the present study. Similarly, the technical parameters discussed by Rittweger [6] correspond to the first category (*information about the vibration*) of the final list. Deviating from the final list, Rittweger [6] emphasized the physiological effects of WBV (e.g., EMG, skin and muscle perfusion, safety concerns). Some of these aspects were included in the first round of the present study (e.g., *report how it is assured that neck and head are not negatively affected*) or were suggested by experts. However, these aspects did not reach consensus and were only included in the optional list (see Tables 4 and 5).

While safety concerns have been given extensive consideration before [6,23], according to the experts’ ratings, this information may not be generally applicable for WBV reporting guidelines. Consideration of safety is, nevertheless, relevant for WBV study design and the mentioned references may be consulted. However, this is a separate issue requiring more attention and investigation. Thus, recommendations concerning safe parameter settings will not be a part of the upcoming reporting guidelines.

One of the starting points for creating the initial included aspects for the Delphi study were the recommendations by Rauch et al. [1]. Their suggestions are, with few exceptions, consistent with the final list of the present study. Instead of the four categories of the present study, Rauch and colleagues [1] recommend reporting aspects of two categories: (1) items related to the WBV device and (2) items related to study participants.

All aspects discussed in their recommendations concerning the first category [1], except skidding of feet (optional item), assessing skidding (not included), and changes in vibration settings (not included), reached consensus in the present study. The aspect of changes in vibration settings may not be relevant for WBV research per se as (1) changes in the protocol of studies should always be reported [34] and (2) reporting a-priori planned changes of vibration parameters is an inherent part of item 4 (*which frequency (Hz) is used*; Table 6) and other items specifying vibration parameters. Aspects of Rauch and colleagues’ second category [1], which are not directly included in the present study are the explained difficulties related to assessing vibration parameters on the body of participants. Yet, this is incorporated in item 14 of the final list (*how vibration parameters should be measured*; Table 6).

While the results of the present study are in accordance with the 13 recommendations by the team around Rauch [1], the results add further 27 WBV-specific aspects to the recommendations. For example, the information concerning participants and administrations, as well as more specifications concerning information about vibration and the device, such as where and how the vibration parameters are measured. Therefore, the final list (1) is an update on previous recommendations, (2) extends beyond the category *information about vibration*, (3) is approved by experts in the field, and (4) may, thus, serve as the foundation on which reporting guidelines can be established upon.

### Limitations

Based on its advantages and the aim of the study, the utilization of the Delphi method was considered appropriate in order to empirically identify items of importance for reporting guidelines of human WBV studies. Yet, some possible limitations must be acknowledged.

Regarding the response rate, of the 136 originally invited experts, 56 opened and started to fill out the first questionnaire and 51 of these completed the questionnaire (41.2% of the invited experts). However, it remains unclear why some invited experts participated in the study while others did not. Thus, the possibility of non-response bias [61] is conceivable, which may hamper the significance of our results. Plausible explanations for non-responses include, but are not limited to, the reason that (1) invitation e-mails were filtered out by the recipient e-mail providers, (2) the expert forgot to fill in the questionnaire despite reminders, (3) the time requirement of participation was too high, (4) the experts were too occupied, (5) the expert may not render the study as relevant, (6) the potential panel member felt they were lacking expertise, and/or (7) one expert participated on behalf of their research team. Since the reasons for non-response have not been investigated, the possible bias on the results cannot be determined. These considerations also apply to the drop-out over the three rounds since it remains unclear why some experts did not continue to be part of the expert panel. The demographic data of the participants over the three rounds indicates that experts from various geographical and academic backgrounds did not participate in subsequent surveys. There is, however, an indication that more physical therapists from Brazil dropped out (42% of overall drop-out) and dropped out panel members who are physical therapists had either a M.Sc. (2 of 7) or PhD (5 of 7). These observations could indicate a potential bias. Possible explanations could be, but are not limited to, that physical therapists in Brazil (1) have a higher work-load and, thus, less time to respond to the surveys in comparison to the other experts; (2) deem the commencement of reporting guidelines unnecessary; (3) respond for a research team more frequently; (4) felt they have less expertise to answer the questions; or (5) judge the topic irrelevant for their field. The explanations of drop-out cannot be (dis)proven without contacting these experts. However, the drop-out led to only very slight changes in age, experience rate, number of published studies and the other demographic information. Notwithstanding, 72.5% of the 51 experts of the first round participated in the study until the end and continued to be part of the panel and measures were taken to reduce the drop-out rate (i.e., reminder e-mails). This rate is sufficient to achieve a valid Delphi process [62,63].

The second limitation of the Delphi method and the present study is the arbitrary determination of a consensus rate. There is no agreed-upon consensus definition in the scientific literature and is defined differently in various studies utilizing this method ranging from an agreement rate of 51% to 100% [37,39,46]. The rational of the boundary conditions for the present study was based on the probable unattainability of an extremely high agreement rate (e.g., 100%), yet the agreement should be considerably higher than 50%. The disagreement condition was introduced to ensure that an item is not accepted in case of diverging opinions, i.e., if there are substantial numbers of experts agreeing but also disagreeing or a high number of experts refrain from giving a definite rating (i.e., choose “neutral/don’t know”). Items without consensus were not disregarded but suggested as optional items. Thus, some items that may be important are not omitted entirely but the reader has the chance to make his or her own evaluation.

Based on the evolving field of WBV research and the limitations of the present study, future research may potentially re-evaluate and update the presented list once the reporting guidelines have been established.

### Strengths

The expertise and knowledge of the panel is one of the main determinants of the validity of the study [39]. If expertise is limited, the results may not reflect the opinion of the scientific community and important aspects may be neglected. One indication of expertise are the characteristics of the panel. For the present study, the characteristics of the experts indicate that they indeed created a panel with expertise and valuable insights in the utilization of WBV, even when considering drop-out. With most panel members having a doctor title (57%) or being a professor (41%), the academic level of the experts was high. Also, the number of publications (*M* = 11.6 in round 3) and years of experience with WBV in research (*M* = 19.4 in round 3) and clinical settings (*M* = 11.6 in round 3) supports the presence of expertise. The inclusion of experts from various geographic locations and academic backgrounds further points to a heterogeneous, experienced expert panel, which increases the quality of responses, as more alternatives are likely to be considered [39,64]. Even though the panel appears to consist of experts of WBV research, their level of expertise of all aspects of WBV may vary. This may explain the high neutrality ratings of the items of no consensus (Tables 4 and 5).

Small panel sizes and response rates may distort validity of the results of a Delphi study. There is little data on panel size and its effect on validity of reaching consensus [64]. Yet, the reliability is thought to increase with panel size, while differences may be small with a panel of more than 12 experts [64]. The present study included 37 to 51 experts, exceeding this advised minimum group size. Additionally, the experts’ characteristics remained diverse regarding age, gender, and geographic location throughout the rounds. One may therefore conclude that the panel size was adequate to collect information and base conclusions on the panel’s expertise [37].

## Conclusion

The present study aimed to commence the establishment of reporting guidelines for human WBV studies based on an empirical inquiry. A list of 40 aspects which were judged to be important in reporting WBV studies (94.6% agreement of the expert panel) was created. This study represents and includes a wide range of experts from various academic and geographic backgrounds and extends the literature to aspects of other important categories, such as *information about the administration*. Thus, the study adds to the existing literature by creating a list of approved-upon items. Furthermore, the guidelines that will be based on the study will extend the general reporting guidelines (e.g., CONSORT statement [34]) for clinical trials and acts as an addition to it. The need of the reporting guidelines, the high agreement on the final list, generally praising comments from the experts, and a low drop-out rate indicate the relevance and importance of the present study.

## Supporting information

S1 File Overview of the executive group. (DOCX)

S2 File All questionnaires of the three rounds. (DOCX)

S3 File Quantitative analysis. (DOCX)
