# Supplementary material for: Towards reporting guidelines of research using whole-body vibration as training or treatment regimen in human subjects—A Delphi consensus study
Source: PLoS One. 2020 Jul 22;15(7):e0235905. doi: 10.1371/journal.pone.0235905 (PMC7375612; doi:10.1371/journal.pone.0235905)
Supplement: S1 File — (DOCX) [file pone.0235905.s001.docx]

Executive Group – Towards Reporting Guidelines in Human WBV Studies

| Name | Affiliation | Discipline | Country |
| --- | --- | --- | --- |
| Mario Bernardo-Filho | Universidade do Estado do Rio de Janeiro | Biomedicine | Brazil |
| Anselm B.M. Fuermaier | University of Groningen | Neuropsychology | the Netherlands |
| Marieke J.G. van Heuvelen | University of Groningen/University Medical Center Groningen | Human Movement Sciences | the Netherlands |
| Stefan Judex | Stony Brook University | Biomedical Engineering | USA |
| Pedro J. Marín | CyMO Research  Institute | Neuromuscular  analysis | Spain |
| Csaba Nyakas | Semmelweis  University Budapest | Neurobiology | Hungary |
| Jörn Rittweger | German Aerospace  Center; University  of Cologne | Space Physiology | Germany |
| Danubia C. Sá-Caputo | Universidade do Estado do Rio de Janeiro | Physiotherapy | Brazil |
| Eckhard Schoenau | University of  Cologne | Pediatrics | Germany |
| Adérito Seixas | Escola Superior de Saúde, Universidade Fernando Pessoa, Porto | Physiotherapie | Portugal |
| Christina Stark | University of  Cologne | Physiotherapy | Germany |
| Redha Taiar | University of Reims | Biomechanics | France |
| Oliver M. Tucha | University of Groningen | Neuropsychology | the Netherlands |
| Eddy A. van der Zee | University of Groningen | Neurobiology | the Netherlands |
