# Supplementary material for: Towards reporting guidelines of research using whole-body vibration as training or treatment regimen in human subjects—A Delphi consensus study
Source: PLoS One. 2020 Jul 22;15(7):e0235905. doi: 10.1371/journal.pone.0235905 (PMC7375612; doi:10.1371/journal.pone.0235905)
Supplement: S2 File — (DOCX) [file pone.0235905.s002.docx]

**Supporting information S1**

**Online questionnaire of all three rounds**

**Round 1**

*Information Sheet*

*Aim of the study*

The central aim of this study is to create reporting guidelines for all disciplines applying whole-body vibration (WBV) within human studies. No agreed-upon reporting systems exists, which lead to misunderstandings and inaccurately provided information in some studies. Focus of this study is, thus, to collect the features specific to WBV studies, which are necessary to ensure high-quality reporting and comparison of studies.

The aim is **not** to collect aspects of studies relevant to reporting research which are outlined in general reporting guidelines (e.g. the CONSORT statement) and are not specific for WBV research (e.g. sample size, age of participants, type of study, or control group intervention).

*Participants of the study*

The study will be based on the opinion of panel experts from different disciplines. All experts have a background of conducting research with WBV. Therefore, researchers who published at least 2 peer-reviewed articles in English utilizing WBV with human subjects are contacted. In total 135 panel experts are invited to share their insights of WBV research to establish these reporting guidelines.

*Procedure*

This online study is expected to consist of approximately three rounds of questionnaires. The completion of each questionnaire will take approximately 5 to 10 minutes and will be online based. You will receive a link to the questionnaire via e-mail. It is expected that the questionnaire rounds will be concluded in four to five months and with that the study is finalized. Throughout the three questionnaires, you will be asked to give your evaluation regarding the importance of several WBV related aspects and indicate more aspects you consider relevant. Before each questionnaire you will be informed of its purpose and main question.

*Confidentiality of research information*

The research results of this study will be treated confidentially and anonymously. Round two and three will start out with summaries of the anonymous group outcomes of the previous rounds. Thus, only the Delphi Steering Group will have access to the personal data during the study. After the completion of all questionnaires, your personal data, like name and age, will be processed by means of a participant number.  Your data will, thus, be processed confidentially, and the result cannot be retraced to you.

*Further information*

If you have questions or suggestions about this study, you may contact the Delphi Steering Group: Anika Wüstefeld [1] (a.wustefeld@student.rug.nl), Dr. Anselm B.M. Fuermaier [1] (a.b.m.fuermaier@rug.nl), Prof. Dr. Oliver M. Tucha [1] (o.m.tucha@rug.nl), Dr. Marieke J.G. van Heuvelen [2] (m.j.g.van.heuvelen@umcg.nl), or Dr. Eddy A. van der Zee [3] (e.a.van.der.zee@rug.nl).

The study has been approved by the Ethical Committee of the Department of Psychology of the University of Groningen, Faculty of Behavioural and Social Sciences, University of Groningen, Grote Kruisstraat 2/1, 9712 TS Groningen, The Netherlands, on the 20/12/2018 (ref. no.; 18211-O).

[1] Department of Clinical and Developmental Neuropsychology, Faculty of Behavioural and Social Sciences, University of Groningen, Grote Kruisstraat 2/1, 9712 TS Groningen, The Netherlands [2] Center for Human Movement Sciences, University of Groningen, University Medical Center Groningen, Antonius Deusinglaan 1, 9713 AV Groningen, The Netherlands [3] Faculty of Science and Engineering, GELIFES — Groningen Institute for Evolutionary Life Sciences, Nijenborgh 7, 9747 AG Groningen, The Netherlands

*Informed Consent*

I hereby consent to be a participant in the current research performed by the Delphi Steering Group.   I have agreed to take part in the study entitled “Towards Reporting Guidelines of Research using Whole-Body Vibration – A Delphi Consensus Study” and I understand that my participation is entirely voluntary. I understand that my responses will be kept strictly confidential and anonymous. I have the option to withdraw from this study at any time, without penalty, and I also have the right to request that my responses will not be used.  
The following points have been explained to me:  
1. The goal of this study is to create guidelines concerning the reporting of all human studies using whole-body vibration (WBV). Participation in this study should help advance high-quality reporting of WBV studies.  
2. I shall be asked to complete some questionnaires indicating the importance of WBV study aspects in several rounds.  
3. This study (~ three questionnaires) will last approximately 30 minutes, with each round taking around 10 minutes.  
4. My responses will be treated confidentially and my anonymity will be ensured. Hence, my responses cannot be identifiable and linked back to me as an individual.  
5. The researcher will answer any questions I might have regarding this research, now or later in the course of the study.

Agree (_)

Do not agree (_)

*General Information*

What is your age in years? ______________________________________________

Please select your gender

- Male
- Female
- Other

Where are you located?

| Country _________________________________  Place of residence _________________________________ |
| --- |

What is your academic background (e.g. psychology, medicine)?

________________________________________________________________

Which is your highest academic degree/position? _______

- B.Sc.
- M.Sc.
- PhD
- Prof

How many years of research experience do you have?

________________________________________________________________

How many years of clinical experience in general do you have?

________________________________________________________________

How do you currently distribute your working time into (1) Clinical work, (2) Research, (3) Other tasks in %?

Clinical work: _______

Research: _______

Other Tasks: _______

Total: ________

How many English, peer-reviewed articles utilizing whole-body vibration (WBV) did you publish so far?

________________________________________________________________

Do you have clinical experience with the use of WBV?

- Yes
- No

*Information about the vibration*

When considering human studies using whole-body vibration is it important to report...

|  | Yes | No | Neutral/Don't know |
| --- | --- | --- | --- |
| ...the type of vibration (i.e. synchronous, side-alternating, other)? |  |  |  |
| ...which frequency (Hz) is used? |  |  |  |
| ...whether the vibration is immediately full or increases slowly? |  |  |  |
| ...whether the frequency (Hz) is constant or variable? |  |  |  |
| ...which peak-to-peak displacement (PDD) of the vibration occurs? |  |  |  |
| ...the amplitude of the vibration (in mm)? |  |  |  |
| ...which peak-acceleration (in multiples of g) occurs? |  |  |  |
| ...which peak-acceleration (in RMS) occurs? |  |  |  |
| ...the units of the vibration parameters? |  |  |  |
| ...the definitions/explanations of the vibration parameters? |  |  |  |
| ...whether manufacturer settings or own settings are used? |  |  |  |
| ...how the vibration parameters are measured, if own settings are used? |  |  |  |
| ...where on the platform the vibration parameters are measured, if own setting are used? |  |  |  |
| ...the position of the platform where PDD or amplitude is measured? |  |  |  |
| ...the position of each foot on the base of the vibration platform to calculate PDD or amplitude for side-alternating vibration? |  |  |  |
| ...what the accuracy of vibration parameters is? |  |  |  |

*Display This Question:*

*If When considering human studies using whole-body vibration is it important to report... = ...how the vibration parameters are measured, if own settings are used? [Yes]*

In your opinion, how should vibration parameters be measured if individual settings are utilized?

________________________________________________________________

*Additional suggestions - Information about vibration*

In your opinion, are there any additional aspects specific to whole-body vibration studies, which were not mentioned with regard to information about the vibration or do you have other questions/comments?

- Yes
- No

*Display This Question:*

*If In your opinion, are there any additional aspects specific to whole-body vibration studies, which... = Yes*

Please list these additional aspects.

________________________________________________________________

________________________________________________________________

________________________________________________________________

*Information about device*

When considering human studies using whole-body vibration is it important to report...

|  | Yes | No | Neutral/Don't know |
| --- | --- | --- | --- |
| ...whether the device vibrates horizontal, vertical, side-alternating, waveform changing, or other? |  |  |  |
| ...the manufacturer, device specifications, and production type? |  |  |  |
| ...the size of the vibration platform? |  |  |  |
| ...whether a handrail is available? |  |  |  |
| ...whether changes are made to the device (e.g. mounting a chair on it)? |  |  |  |
| ...how the energy is generated (e.g. direct mechanical transmission or electromagnetic transmission) as this can have an effect on the performance of the device? |  |  |  |

*Additional suggestions – Information about device*

In your opinion, are there any additional aspects specific to whole-body vibration studies, which were not mentioned with regard to information about the used device or do you have other questions/comments?

- Yes
- No

*Display This Question:*

*If In your opinion, are there any additional aspects specific to whole-body vibration studies, which... = Yes*

Please list these additional aspects.

________________________________________________________________

________________________________________________________________

________________________________________________________________

________________________________________________________________

________________________________________________________________

*Information about administration*

When considering human studies using whole-body vibration is it important to report...

|  | Yes | No | Neutral/Don't know |
| --- | --- | --- | --- |
| ...where the feet of the participants are placed? |  |  |  |
| ...where the hands of the participants are placed? |  |  |  |
| ...which posture or body position the participants take on during the vibration (e.g. sitting, standing, squatting)? |  |  |  |
| ...whether the position/posture changes during the WBV (static versus dynamic exercise)? |  |  |  |
| ...which exact tools and aids were used during the WBV (e.g. type and size of dumbbells)? |  |  |  |
| ...how resonance (skidding of feet) is prevented (e.g. front-foot vs. mid-foot stance)? |  |  |  |
| ...the number of sessions where WBV was utilized? |  |  |  |
| ...the resting time between sessions of WBV? |  |  |  |
| ...the total exposure time to WBV across all sessions? |  |  |  |
| ...the number of exposures to WBV within one session? |  |  |  |
| ...the on vs. off-times of vibration (e.g. pauses and how long) within one session? |  |  |  |
| ...whether the cognitive/physical outcome measures are assessed during or after the WBV? |  |  |  |
| ...the conditions of the test room (e.g. light and temperature)? |  |  |  |
| ...the location of the intervention (e.g. hospital or gym)? |  |  |  |
| ...whether an examiner was present to supervise the WBV administration? |  |  |  |
| ...possible follow-ups to determine possible lasting effects? |  |  |  |

*Additional suggestions – Information about administration*

In your opinion, are there any additional aspects specific to whole-body vibration studies, which were not mentioned with regard to information about the administration do you have other questions/comments?

- Yes
- No

*Display This Question:*

*If In your opinion, are there any additional aspects specific to whole-body vibration studies, which... = Yes*

Please list these additional aspects.

________________________________________________________________

________________________________________________________________

________________________________________________________________

________________________________________________________________

________________________________________________________________

*Information about participants*

When considering human studies using whole-body vibration is it important to report...

|  | Yes | No | Neutral/Don't know |
| --- | --- | --- | --- |
| ...how it is assured that neck and head are not negatively affected by the vibration? |  |  |  |
| ...the participants' BMI (height, weight)? |  |  |  |
| ...whether the participants wore glasses during WBV? |  |  |  |
| ...whether possible glasses were disturbing during the vibration? |  |  |  |
| ...the participants' footwear (shoes, socks, barefoot) during WBV with a detailed description? |  |  |  |
| ...the participants' fitness and activity levels? |  |  |  |
| ...skin and muscle perfusion during the WBV? |  |  |  |
| ...the participants' clothing during the WBV? |  |  |  |
| ...if, how, and for how long participants prepared for the WBV (e.g. stretching, muscles, warm up)? |  |  |  |
| ...subjective experiences of participants before, during, or after the WBV (e.g. dizziness, pain, postural instability, muscle fatigue)? |  |  |  |

*Display This Question:*

*If When considering human studies using whole-body vibration is it important to report... = ...subjective experiences of participants before, during, or after the WBV (e.g. dizziness, pain, postural instability, muscle fatigue)? [Yes]*

In your opinion, which subjective experiences of participants should be reported?

________________________________________________________________

________________________________________________________________

________________________________________________________________

*Additional suggestions – Information about participants*

In your opinion, are there any additional aspects specific to whole-body vibration studies, which were not mentioned with regard to information about the participants do you have other questions/comments?

- Yes
- No

*Display This Question:*

*If In your opinion, are there any additional aspects specific to whole-body vibration studies, which... = Yes*

Please list these additional aspects.

________________________________________________________________

________________________________________________________________

________________________________________________________________

________________________________________________________________

________________________________________________________________

*Comments/Suggestions*

Do you have any other comments or suggestions not previously stated concerning the study or its contents?

________________________________________________________________

________________________________________________________________

________________________________________________________________

________________________________________________________________

________________________________________________________________

**End of Questionnaire 1**

**Round 2**

*Information Sheet*

Welcome back to the second round of the Delphi Consensus Study building towards reporting guidelines of human whole-body vibration studies.

 *Aim of the second round*
 As part of the panel of 51 experts from 17 countries, you will be asked to rate the items of the previous round again. This will be divided into the four thematic blocks: information about vibration, information about the device, information about the administration, and information about the participants.
Along with the items (e.g. 'report the posture of the participants') you will see the agreement/disagreement/neutral rating of the previous round (e.g. 25/25/50 in %). Combining your valued opinion and the panel's rating, you will be asked again to indicate whether the items should be reported in human WBV studies.
Also, additional items, as suggested by the expert panel during the first round, will be presented. You will be asked to rate these and indicate your esteemed opinion. For example, an additionally suggested item 'report the footwear of the participants' will be presented together with how many of the experts suggested this item (e.g. 15 of 51).

In the previous round we received additional comments regarding further literature and general demographic aspects. Since the aim of this study is to achieve consensus regarding aspects that are specific to whole-body vibration studies, these aspects will not be further considered. However, demographics and further literature will be discussed in the final report.
  
We, the Delphi Steering Group, would like to thank you for your participation in the first round and are looking forward to continuing to integrate your valued opinion with the participation in the second round. 
 
The Delphi Steering Group
Anika Wüstefeld, Dr. Anselm B.M.Fuermaier, Dr. Marieke J.G. van Heuvelen, Prof Dr. Eddy A. van der Zee, and
 Prof. Dr. Oliver M.Tucha
 
For further questions please contact Anika Wustefeld (a.wustefeld@student.rug.nl)

**I. Information about the vibration**

In the following block you will see the items referring to information about the vibration. The expert panel's ratings of the first round (in % of agreement/disagreement/neutral) will be shown along with the items.

Please rate these items again with regard to the importance of reporting the information in human WBV studies, while taking the opinion of the panel into account.
The previous agreement of the panel members is presented in % per item: Agree/Disagree/Neutral

|  | Agree | Disagree | Neutral/Don't know |
| --- | --- | --- | --- |
| ...which frequency (Hz) is used? 100/0/0 |  |  |  |
| ...whether the frequency is constant or variable? 98/0/2 |  |  |  |
| ...the type of vibration (i.e. synchronous, side-alternating, other)? 96/4/0 |  |  |  |
| ...the units of the vibration parameters? 94/4/2 |  |  |  |
| ...the amplitude of the vibration (in mm)? 94/2/4 |  |  |  |
| ...how the vibration parameters are measured? 86/4/10 |  |  |  |
| ...the definitions/explanations of the vibration parameters? 80/14/6 |  |  |  |
| ...which peak-acceleration (in multiples of g) occurs? 80/6/14 |  |  |  |
| ..the position of each foot on the base of the vibration platform to calculate PDD/amplitude for side-alternating vibration)? 80/4/16 |  |  |  |
| ...whether manufacturer settings or own settings are used? 78/12/10 |  |  |  |
| ...whether vibration is immediately full or increases slowly? 78/10/12 |  |  |  |
| ...which peak-to-peak displacement (PDD) of the vibration occurs? 78/8/14 |  |  |  |
| ...where on the platform the vibration parameters are measured? 75/6/20 |  |  |  |
| ...what the accuracy of the vibration parameters is? 69/8/24 |  |  |  |
| ...which peak-acceleration occurs based on pilot study or relevant literature? 37/24/39 |  |  |  |

In the following you will see *additionally suggested items* of the first round referring to information about the vibration.

Please rate these items with regard to their importance of reporting them in human WBV studies.
 The number of times suggested is indicated behind each item in [_].

|  | Agree | Disagree | Neutral/Don't know |
| --- | --- | --- | --- |
| ...sEMGs (surface electromyography) to evaluate and record the electrical signals produced by muscle activity [2x] |  |  |  |
| ...how the subject's body mass affects the vibration parameters (compare measure with and without subject on the platform)? [2x] |  |  |  |
| ...the intensity of the exposure (in m/s^2 RMS)? [1x] |  |  |  |
| ...the vertical and horizontal accelerations? [1x] |  |  |  |
| ...report whether manufacturer settings are used *but* additionally report own vibration parameter measurements? [2x] |  |  |  |
| ...if manufacturer settings are used, is it sufficient to report these? [1x] |  |  |  |
| ...always measure vibration parameters, even if manufacturer settings are used? [1x] |  |  |  |

In the first round, you have been asked *how vibration parameters should be measured*. In the following you will be asked to rate the suggestions made by the expert panel.

The number of times suggested by the expert panel is indicated behind the items in [_].

Measure vibration parameters with accelerometers [24x]

- Agree
- Disagree
- Neutral/Don't know

*Display This Question:*

*If Measure vibration parameters with accelerometers [24x] = Agree*

How should the accelerometers be utilized? Please select one of the options.

- 3D-accelerometer for vertical and horizontal acceleration
- Accelerometer at vibration platform only
- Accelerometer on participant only (e.g. at joints)
- Accelerometer at vibration platform and on participant
- It does not matter how they are measured, as long as they are
- Other, namely ________________________________________________

Further suggestions about *measuring vibration parameters* were recorded in the first round. Please indicate whether you agree with the importance of reporting these suggestions in human WBV studies.

The number of times suggested is indicated behind each item in [_].

|  | Agree | Disagree | Neutral/Don't know |
| --- | --- | --- | --- |
| Frequency and amplitude [4x] |  |  |  |
| Frequency and amplitude over time [1x] |  |  |  |
| Frequency and amplitude including RMS (in multiples of g) [1x] |  |  |  |
| Frequency, amplitude, and peak-to-peak displacement [1x] |  |  |  |
| Laser Distance Sensor [2x] |  |  |  |
| Peak-to-peak amplitude rather than RMS [1x] |  |  |  |
| Gyroscope [1x] |  |  |  |
| It is not important how they are measured but that they are reported [1x] |  |  |  |
| Vibration parameters should be measured to the individual case [1x] |  |  |  |

**II. Information about the device**

In the following block you will see the items referring to information about the device. The expert panel's ratings of the first round (in % of agreement/disagreement/neutral) will be shown along with the items.

Please rate these items again with regard to their importance of reporting them in human WBV studies, while taking the opinion of the panel into account.

The previous agreement of the panel members is presented in % per item: Agree/Disagree/Neutral

|  | Agree | Disagree | Neutral/Don't know |
| --- | --- | --- | --- |
| ...whether the device vibrates horizontal, vertical, side-alternating, waveform changing, or other? 98/2/0 |  |  |  |
| ...whether changes are made to the device (e.g. mounting a chair on it)? 96/0/4 |  |  |  |
| ...the manufacturer, device specifications, and production type? 94/4/2 |  |  |  |
| ...whether a handrail is available? 82/10/8 |  |  |  |
| ...how the energy is generated (e.g. direct mechanical transmission or electromagnetic transmission) as this can have an effect on the performance of the device? 55/8/37 |  |  |  |
| ...the size of the vibration platform? 49/24/27 |  |  |  |

**III. Information about the administration**

In the following block you will see the items referring to information about the administration. The expert panel's ratings of the first round (in % of agreement/disagreement/neutral) will be shown along with the items.

Please rate these items again with regard to their importance of reporting them in human WBV studies, while taking the opinion of the panel into account.
The previous agreement of the panel members is presented in % per item: Agree/Disagree/Neutral

|  | Agree | Disagree | Neutral/Don't know |
| --- | --- | --- | --- |
| ...which posture or body position the participants take on during the vibration (e.g. sitting, standing, squatting)? 100/0/0 |  |  |  |
| ...whether the position/posture changes during the WBV (static versus dynamic exercise)? 100/0/0 |  |  |  |
| ...the number of sessions where WBV was utilized? 100/0/0 |  |  |  |
| ...the resting time between sessions of WBV? 100/0/0 |  |  |  |
| ...the number of exposures to WBV within one session? 98/0/2 |  |  |  |
| ...where the feet of the participants are placed? 96/0/4 |  |  |  |
| ...the total exposure time to WBV across all sessions? 94/4/2 |  |  |  |
| ...where the hands of the participants are placed? 92/2/6 |  |  |  |
| ...which exact tools and aids were used during the WBV (e.g. type and size of dumbbells)? 90/2/8 |  |  |  |
| ...the on vs. off-times of vibration (e.g. pauses and how long) within one session? 88/2/10 |  |  |  |
| ...whether an examiner was present to supervise the WBV administration? 84/10/6 |  |  |  |
| ...whether the outcome measures (cognitive/physical) are assessed during or after the WBV? 80/10/10 |  |  |  |
| ...possible follow-ups to determine possible lasting effects? 80/6/14 |  |  |  |
| ...how resonance (skidding of feet) is prevented (e.g. front-foot vs. mid-foot stance)? 76/6/18 |  |  |  |
| ...the location of the intervention (e.g. hospital, home, gym)? 59/25/16 |  |  |  |
| ...the conditions of the test room (e.g. light and temperature)? 47/33/20 |  |  |  |

In the following you will see *additionally suggested items* of the first round referring to information about the administration.

Please rate these items with regard to their importance of reporting them in human WBV studies.

The number of times suggested is indicated behind each item in [_].

|  | Agree | Disagree | Neutral/Don't know |
| --- | --- | --- | --- |
| ...to report whether only parts of the subjects' body are subjected to vibration (e.g. only the feet)? [2x] |  |  |  |
| ...to explain the decision which parts (e.g. only feet) of the participants are subjected to vibration and why [1x] |  |  |  |

**IV. Information about the participants**

In the following block you will see the items referring to information about the participants. The expert panel's ratings of the first round (in % of agreement/disagreement/neutral) will be shown along with the items.

Please rate these items again with regard to their importance of reporting them in human WBV studies, while taking the opinion of the panel into account.

The previous agreement of the panel members is presented in % per item: Agree/Disagree/Neutral

|  | Agree | Disagree | Neutral/Don't know |
| --- | --- | --- | --- |
| ...subjective experiences of participants before, during, or after the WBV (e.g. dizziness, pain, postural instability, muscle fatigue)? 90/2/8 |  |  |  |
| ...the participants' height and weight? 88/8/4 |  |  |  |
| ...the participants' footwear (shoes, socks, barefoot) during WBV with a detailed description? 88/4/8 |  |  |  |
| ...if, how, and for how long participants prepared for the WBV (e.g. stretching, muscles, warm up)? 84/4/12 |  |  |  |
| ...the participants' fitness and activity levels? 84/2/14 |  |  |  |
| ...how it was assured that neck and head are not negatively affected by the vibration? 65/16/20 |  |  |  |
| ...the participants' clothing during the WBV? 47/29/24 |  |  |  |
| ...skin and muscle perfusion during the WBV? 31/39/29 |  |  |  |
| ...whether possible glasses were disturbing during the vibration? 27/33/39 |  |  |  |
| ...whether the participants wore glasses during WBV? 25/39/35 |  |  |  |

In the following you will see *additionally suggested items* of the first round referring to information about the participants*.*

Please rate these items with regard to their importance of reporting them in human WBV studies.
 The number of times suggested is indicated behind each item in [_].

|  | Agree | Disagree | Neutral/Don't know |
| --- | --- | --- | --- |
| Training history/activity levels [3x] |  |  |  |
| History of injuries [1x] |  |  |  |

In the first round, you have been asked *which subjective experiences should be reported*. In the following you will be asked to rate whether these subjective experiences should be reported in WBV studies.

The number of times suggested by the expert panel is indicated behind the items in [_].

|  | Agree | Disagree | Neutral/Don't know |
| --- | --- | --- | --- |
| Pain (location, intensity, type) [16x] |  |  |  |
| Dizziness [12x] |  |  |  |
| Fatigue/exhaustion/tiredness [8x] |  |  |  |
| Tingling/itching/burning sensations [8x] |  |  |  |
| Perceived exertion/effort (e.g. with Borg RPE scale) [5x] |  |  |  |
| (Dis)comfort [3x] |  |  |  |
| Subjective experience depends on the aim of the study and cannot be generalized [3x] |  |  |  |
| Side effects/adverse effects [2x] |  |  |  |
| Muscle soreness/weakness/cramps [2x] |  |  |  |
| Headache [2x] |  |  |  |
| Loss of balance [2x] |  |  |  |
| Redness [2x] |  |  |  |
| Vertigo [2x] |  |  |  |
| Enjoyment [1x] |  |  |  |
| Nausea [1x] |  |  |  |
| Mood [1x] |  |  |  |

**End of Questionnaire 2**

**Round 3**

*Information Sheet*

Welcome back to the final round of the Delphi Consensus Study building towards reporting guidelines of human whole-body vibration studies.
  
*Aim of the final round*
After the two previous rounds with a panel of 40 experts, consensus for certain items which should be reported in human WBV studies has been obtained. Per item, we consider consensus to be an agreement-rate of 70% or higher while having a disagreement-rate below 20%. In the following, you will receive the final list of items for which consensus has been reached along with their final ratings.

You, as part of the expert panel, will be asked to indicate whether you agree with this final list. While some of the items with consensus may not be applicable to your own studies, it is important to consider that they may be relevant for other studies. If you do not agree with the final list, we would appreciate if you indicate the reason for your disagreement (i.e. with which aspect of this list you disagree).  
 As mentioned before, aspects that are relevant for all studies (e.g. demographics of participants) and further literature will be discussed in the final report.
  
We, the Delphi Steering Group, would like to thank you again for participating in the previous rounds and are looking forward to continuing to establish reporting guidelines based on your valued opinion. 
 
The Delphi Steering Group
Anika Wustefeld, Dr. Anselm B.M. Fuermaier, Dr. Marieke J.G. van Heuvelen, Prof Dr. Eddy A. van der Zee, and Prof. Dr. Oliver M.Tucha
 
For further questions please contact Anika Wustefeld (a.wustefeld@student.rug.nl)

*Final List*

In the following you see the final list of items which should be reported in human WBV studies based on the previous questionnaires. The list is divided into the previously introduced four categories (1) vibration, (2) the device, (3) the administration, and (4) the participants. Additionally, you can see the ratings of the previous round.

**Please read through the list and indicate whether you agree or disagree with these final reporting guidelines.**

When considering human studies using whole-body vibration it is important to report (% of Agreement/Disagreement/Neutral)

 *I. Information about vibration*
 1. the type of vibration (i.e. synchronous, side-alternating, other) (100/0/0)
 2. the units of the vibration parameters (100/0/0)
 3. the amplitude of the vibration (in mm) (100/0/0)
 4. which frequency (Hz) is used (97.5/2.5/0)
 5. the position of each foot on the base of the vibration platform to calculate PDD or amplitude for side-alternating vibration (92.5/2.5/5)
 6. whether the frequency (Hz) is constant or variable (92.5/7.5/0)
 7. whether manufacturer settings or own settings are used (87.5/5/5)
 8. how the vibration parameters are measured (85/7.5/7.5)
 9. whether the vibration is immediately full or increases slowly (85/10/7.5)
 10. which peak-to-peak displacement (PDD) of the vibration occurs (85/7.5/12.5)
 11. where on the platform the vibration parameters are measured (82.5/5/7.5)
 12. which peak-acceleration (in multiples of g) occurs (80/7.5/12.5)
 13. the definitions/explanations of the vibration parameters (77.5/7.5/15)
 *Vibration parameters should be measured with*
 14. with frequency and amplitude (92.5/2.5/5), or
 15. the aid of accelerometers (77.5/15/7.5) either with an accelerometer at vibration platform and on participant (45.2%) and/or 3D-accelerometer for vertical and horizontal acceleration (32.5%), or
 16. with frequency, amplitude, and peak-to-peak displacement (70/10/20)

 *II. Information about device*
 1. whether the device vibrates horizontal, vertical, side-alternating, waveform changing, or other (100/0/0)
 2. whether changes are made to the device (e.g. mounting a chair on it) (97.5/0/2.5)
 3. the manufacturer, device specifications, and production type (97.5/0/2.5)
 4. whether a handrail is available (85/7.5/7.5)

 *III. Information about administration*
 1. which posture or body position the participants take on during the vibration (e.g. sitting, standing, squatting) (100/0/0)
 2. whether the position/posture changes during the WBV (static versus dynamic exercise) (100/0/0)
 3. the number of sessions where WBV was utilized (100/0/0)
 4. the resting time between sessions of WBV (100/0/0)
 5. the number of exposures to WBV within one session (97.5/0/2.5)
 6. where the feet of the participants are placed (97.5/0/2.5)
 7. the total exposure time to WBV across all sessions (97.5/2.5/0)
 8. where the hands of the participants are placed (92.5/5/2.5)
 9. the on vs. off-times of vibration (e.g. pauses and how long) within one session (92.5/5/2.5)
 10. which exact tools and aids were used during the WBV (e.g. type and size of dumbbells) (90/2.5/7.5)
 11. whether the outcome measures (cognitive/physical) are assessed during or after the WBV (87.5/7.5/5)
 12. whether an examiner was present to supervise the WBV administration (85/10/5)
 13. possible follow-ups to determine possible lasting effects (85/10/5)
 14. to report whether only parts of the subjects' body are subjected to vibration (e.g. only the feet) (82.5/7.5/10)
 15. the location of the intervention (e.g. hospital or gym) (70/12.5/17.5)
 16. to explain the decision which parts (e.g. only feet) of the participants are subjected to vibration and why (70/20/10)

 *IV. Information about participants*
 1. the participants' height and weight (97.5/2.5/0)
 2. the participants' footwear (shoes, socks, barefoot) during WBV with a detailed description (95/2.5/2.5)
 3. subjective experiences of participants before, during, or after the WBV (92.5/5/2.5), which are:
     I. Side effects/adverse effects (100/0/0)
     II. Pain (90/2.5/7.5)
     III. Dizziness (87.5/5/7.5)
     IV. (Dis)comfort (82.5/7.5/10)
     V. Fatigue/exhaustion/tiredness (80/7.5/12.5)
     VI. Tingling/itching/burning sensations (80/2.5/17.5)
     VII. Perceived exertion/effort (e.g. with Borg RPE scale) (77.5/7.5/15)
     VIII. Muscle soreness/weakness (75/7.5/17.5)
     IX. Headache (75/7.5/17.5)
     X. Loss of balance (72.5/10/17.5)
 4. the participants' fitness and activity levels (92.5/2.5/5)
 5. if, how, and for how long participants prepared for the WBV (e.g. stretching, muscles, warm up) (90/2.5/7.5)
 6. training history (82.5/10/10)

Do you agree with this final list?

 Please keep in mind that even though some items might not generally apply to all studies, they still may be relevant to report for specific types of studies.

- Yes
- No

*Display This Question:*

*If Do you agree with this final list? Please keep in mind that even though some items might not gene... = No*

You indicated you disagree with the final list.

Please specify as detailed as possible why you disagree (e.g. which certain item you disagree with).

________________________________________________________________

________________________________________________________________

________________________________________________________________

________________________________________________________________

________________________________________________________________

**End of Questionnaire 3**
