# Supplementary material for: Towards reporting guidelines of research using whole-body vibration as training or treatment regimen in human subjects—A Delphi consensus study
Source: PLoS One. 2020 Jul 22;15(7):e0235905. doi: 10.1371/journal.pone.0235905 (PMC7375612; doi:10.1371/journal.pone.0235905)
Supplement: S3 File — (DOCX) [file pone.0235905.s003.docx]

Quantitative Analysis of the Results

**Method**

The data was analyzed with the statistical programming language R [1] and additional packages to compute Krippendorff’s alpha [2] and McNemar’s chi-square test [3].

Krippendorff’s alpha [4] is a measure of agreement, which is more flexible than other similar measures, such as Cohen’s Kappa or Fleiss’ Kappa [4-6]. This estimate can be utilized for more than two raters and can deal with missing data if two or more observations are available per item. Krippendorff’s alpha compares observed disagreement with expected disagreement and values range from 0 to 1. In case of extreme (dis)agreement, the expected disagreement is influenced by the ratio of agreement/disagreement values and adjusts accordingly. This leads to a low reliability statistic even though the percent agreement is high (so-called κ-Paradox; e.g., in [7-9]).

McNemar’s χ²-test is used to quantify the changes in rating between round 1 and round 2 [10,11]. It is a modified paired-*t* test which can be utilized for categorical data [12]. Since round 3 constitutes the final rating of the whole generated list of items (i.e., post-group consensus [13]), in comparison to the rating of each individual item, the shift in responses is not of interest for round 2 to round 3. The McNemar test requires a minimum of number of observations in each contingency table cell and requires an observation in concordant cells (e.g., ‘neutral’/’neutral’), which is why this statistic may only be computed for items with lower agreement rates and varying responses. Additionally, assessing a shift from, for example 98% agreement to 100% agreement, does not aid in the interpretation of the results. If the McNemar test results in a significant p-value (α≤.05) it indicates a significant difference in the ratings of importance between round 1 and 2. Additionally, Cohen’s Kappa will be calculated as a measure of agreement between the ratings of round 1 and 2 for each item [14,15].

**Results**

Percent agreement increased between the rounds from 70% to 99% (Table S3.1). Point estimates of Krippendorff’s alpha increased from round 1 to round 2, while the estimate is lowest for round 3.

**Table S3.1. Results of Krippendorff’s alpha estimates for all rounds.**

| **Round** | **% Agreement** | **% Chance Agreement** | **Krippendorff’s α estimate** | **Standard error** | **Krippendorff’s α CI (95%)** | **p-value** |
| --- | --- | --- | --- | --- | --- | --- |
| *1* | .707 | .648 | .167 | 0 | (.168; .168) | 0 |
| *2* | .643 | .554 | .200 | .025 | (.151; .249) | <.01 |
| *3* | .986 | .986 | -.007 | .002 | (-.01; -.003) | <.01 |
| *Overall* | .747 | .671 | .231 | .019 | (.192; .27) | 0 |

For 25 of the 27 items where the McNemar test was computed, no significant shifts in ratings from round 1 to round 2 were observable (Table S3.2). Item 47 (*whether possible glasses were disturbing during the vibration*) and Item 48 (*whether the participants wore glasses during WBV*) showed a significant shift in ratings, both becoming more negative (i.e., favoring excluding these items). Estimates of Cohen’s Kappa indicate slight to moderate agreement between round 1 and round 1 across all items [16].

**Table S3.2. Results of McNemar χ²-Test and Cohen’s Kappa for the Pre-Determined Items rated in Round 1 and 2.**

| **Category** | **Item** | **McNemar’s χ²** | **df** | **p-value** | **Cohen’s Kappa (95%-CI)** |
| --- | --- | --- | --- | --- | --- |
| *Information about vibration* | 1 | - | - | - | ***** |
|  | 2 | .25 | 1 | .617 | ***** |
|  | 3 | - | - | - | ***** |
|  | 4 | - | - | - | ***** |
|  | 5 | - | - | - | ***** |
|  | 6 | 1.333 | 3 | .721 | .44 (.13;.75) |
|  | 7 | 3.086 | 3 | .379 | .07 (-.16; .30) |
|  | 8 | - |  |  | .65 (.39, .90) |
|  | 9 | 5.667 | 2 | .129 | .18 (-.09, .45) |
|  | 10 | 5 | 3 | .172 | .43 (.12; .73) |
|  | 11 | 1.667 | 3 | .644 | .09 (-.18; .37) |
|  | 12 | - | - | - | -.002 (-.26; .26) |
|  | 13 | - | - | - | .58 (.33, .83) |
|  | 14 | 4 | 3 | .262 | .23 (-.06; .52) |
|  | 15 | 3.619 | 3 | .306 | .46 (.22; .71) |
|  | 16 | 5.743 | 3 | .125 | .20 (-.03; .44) |
| *Information about device* | 17 | - | - | - | ***** |
|  | 18 | - | - | - | 1 (1;1) |
|  | 19 | - | - | - | -.29 (-.58; .02) |
|  | 20 | 4 | 3 | .262 | .38 (.07; .7) |
|  | 21 | 4.667 | 3 | .198 | .52 (.30; .73) |
|  | 22 | 2.578 | 3 | .461 | .23 (-.003; .46) |
| *Information about administration* | 23 | - | - | - | ***** |
|  | 24 | - | - | - | ***** |
|  | 25 | - | - | - | ***** |
|  | 26 | - | - | - | ***** |
|  | 27 | 0 | 1 | 1 | -.026 (-.06; .01) |
|  | 28 | 0 | 1 | 1 | -.034 (-.08; .01) |
|  | 29 | - | - | - | -.5 (-1; .19) |
|  | 30 | - | - | - | .24 (-.21; .69) |
|  | 31 | - | - | - | .14 (-.23; .52) |
|  | 32 | - | - | - | .20 (-.20; .60) |
|  | 33 | - | - | - | .29 (-.09; .67) |
|  | 34 | 3.333 | 3 | .343 | .48 (.15; .8) |
|  | 35 | 3.286 | 3 | .349 | .16 (-.09; .42) |
|  | 36 | 3.111 | 3 | .375 | .24 (-.03; .51) |
|  | 37 | 4.5 | 3 | .212 | .29 (.04; .55) |
|  | 38 | 1.667 | 3 | .644 | .36 (.13; .59) |
| *Information about participants* | 39 | - | - | - | .24 (-.21; .69) |
|  | 40 | - | - | - | ***** |
|  | 41 | 4 | 3 | .262 | .07 (-.12; .27) |
|  | 42 | 2.667 | 3 | .446 | .19 (-.13; .5) |
|  | 43 | 5.571 | 3 | .134 | .02 (-.14; .18) |
|  | 44 | 2.8 | 3 | .424 | .41 (.18; .64) |
|  | 45 | 1.343 | 3 | .719 | .19 (-.03; .42) |
|  | 46 | 4.971 | 3 | .174 | .19 (-.03; .42) |
|  | 47 | 8.273 | 3 | .041 | .36 (.13; .59) |
|  | 48 | 9.571 | 3 | .023 | .28 (.05; .50) |

*Cohen’s Kappa cannot be calculated for items without responses in all cells of the contingency tables.

**Discussion**

Even though Krippfendorff’s alpha estimates appear to be poor in all three rounds and overall, the percent agreement is high (>70%) in all rounds. Additionally, the ratings per item show that agreement for most items was rather stable throughout all rounds, with high agreement ratings for most items starting in round 1. For example, 13 of 16 items of *information about vibration* show an agreement rate of at least 75% in round 1. Thus, it is plausible that the reliability statistics are underestimated. The overall agreement is further included in the final round, where the experts were asked to agree/disagree with the final list of items. Additionally, the overall agreement rate does not dispute the findings of the study. The main interest lies on the agreement of each item individually (i.e., whether this specific item should be included in the final list or not). The agreement between round 1 and 2 (Cohen’s Kappa) appears to be similarly underestimated by Cohen’s Kappa as it indicates low agreement between the rounds. Nevertheless, also for this statistic the measure may not be of high validity as we expect changes in the ratings from round 1 to round 2 based on the re-assessment of each item with the results of the prior round.

The stability over the rounds was assessed with the McNemar test. No significant shift for most items was observed, which indicates a high stability over the rounds and supports that no more than three rounds were required to achieve stability in the ratings of the experts. Two items showed a significant shift of opinion from round 1 to round 2 resulting in high disagreement rates. These items were ultimately excluded from the final list. Many items reached consensus (according to our definition) in the second round without significant shifts in opinion according to the McNemar test. This reflects the difference of the concepts of stability of ratings and consensus [10].

**References**

1. R Core Team. R: A language and environment for statistical computing. . 2014.

2. Gwet KL. irrCAC: Computing Chance-Corrected Agreement Coefficients (CAC). . 2019.

3. Revelle W. psych: Procedures for Psychological, Psychometric, and Personality Research. . 2019.

4. Krippendorff K. Estimating the Reliability, Systematic Error and Random Error of Interval Data. Educational and Psychological Measurement. 1970;30: 61-70.

5. Zapf A, Castell S, Morawietz L, Karch A. Measuring inter-rater reliability for nominal data â€“ which coefficients and confidence intervals are appropriate? BMC Medical Research Methodology. 2016;16: 93.

6. Hayes AF, Krippendorff K. Answering the Call for a Standard Reliability Measure for Coding Data. Communication Methods and Measures. 2007;1: 77-89.

7. Walsh P, Thornton J, Asato J, Walker N, McCoy G, Baal J, et al. Approaches to describing inter-rater reliability of the overall clinical appearance of febrile infants and toddlers in the emergency department. PeerJ. 2014;2: e651-e651.

8. Gwet KL. Computing inter-rater reliability and its variance in the presence of high agreement. Br J Math Stat Psychol. 2008;61: 29-48.

9. Feinstein AR, Cicchetti DV. High agreement but low Kappa: I. the problems of two paradoxes. J Clin Epidemiol. 1990;43: 543-549.

10. Rayens MK, Hahn EJ. Building consensus using the policy Delphi method. Policy, Politics, & Nursing Practice. 2000;1: 308-318.

11. Hahn EJ, Rayens MK. Consensus for tobacco policy among former state legislators using the policy Delphi method. Tob Control. 1999;8: 137.

12. McNemar Q. Note on the sampling error of the difference between correlated proportions or percentages. Psychometrika. 1947;12: 153-157.

13. von der Gracht HA. Consensus measurement in Delphi studies: Review and implications for future quality assurance. Technological Forecasting and Social Change. 2012;79: 1525-1536. doi: https://doi-org.proxy.kib.ki.se/10.1016/j.techfore.2012.04.013.

14. Weir CR, Hicken BL, Rappaport H(, Nebeker JR. Crossing the Quality Chasm: The Role of Information Technology Departments. Am J Med Qual. 2006;21: 382-393.

15. Molnar FJ, Man-Son-Hing M, Dalziel WB, Mitchell SL, Power BE, Byszewski AM, et al. Assessing the quality of newspaper medical advice columns for elderly readers. CMAJ : Canadian Medical Association journal = journal de l'Association medicale canadienne. 1999;161: 393-395.

16. Landis JR, Koch GG. The measurement of observer agreement for categorical data. Biometrics. 1977;33: 159-174.
